# Src Regulation of Cx43 Phosphorylation and Gap Junction Turnover

**DOI:** 10.3390/biom10121596

**Published:** 2020-11-24

**Authors:** Joell L. Solan, Paul D. Lampe

**Affiliations:** 1 Translational Research Program, Fred Hutchinson Cancer Research Center, Seattle, WA 98109, USA; jsolan@fredhutch.org; 2 Department of Global Health, Pathobiology Program, University of Washington, Seattle, WA 98109, USA

**Keywords:** connexin, gap junction, src, turnover, annular junctions, connexisome

## Abstract

The gap junction protein Connexin43 (Cx43) is highly regulated by phosphorylation at over a dozen sites by probably at least as many kinases. This Cx43 “kinome” plays an important role in gap junction assembly and turnover. We sought to gain a better understanding of the interrelationship of these phosphorylation events particularly related to src activation and Cx43 turnover. Using state-of-the-art live imaging methods, specific inhibitors and many phosphorylation-status specific antibodies, we found phospho-specific domains in gap junction plaques and show evidence that multiple pathways of disassembly exist and can be regulated at the cellular and subcellular level. We found Src activation promotes formation of connexisomes (internalized gap junctions) in a process involving ERK-mediated phosphorylation of S279/282. Proteasome inhibition dramatically and rapidly restored gap junctions in the presence of Src and led to dramatic changes in the Cx43 phospho-profile including to increased Y247, Y265, S279/282, S365, and S373 phosphorylation. Lysosomal inhibition, on the other hand, nearly eliminated phosphorylation on Y247 and Y265 and reduced S368 and S373 while increasing S279/282 phosphorylation levels. We present a model of gap junction disassembly where multiple modes of disassembly are regulated by phosphorylation and can have differential effects on cellular signaling.

## 1. Introduction

Gap junctions are specialized membrane domains composed of tens to thousands of channels that permit intercellular exchange of ions and members of the metabolome of <1000 Da [1,2,3,4,5] (although small RNAs may pass [6,7]). In vertebrates, gap junctions are composed of proteins from the connexin gene family [2,8,9,10]. The basic building block of a gap junction is a connexon which is made of six connexin molecules organized as a hemichannel. Connexons traffic to the plasma membrane where they cluster and form subcellular structures with a distinct morphology that is easily recognizable via transmission and freeze fracture electron microscopy or immunofluorescence in tissue or cells where they have a punctate appearance [11,12,13]. Connexins are expressed in a tissue-specific manner and are critically important in many cellular processes including control of cell migration and proliferation, embryonic development and differentiation, wound repair and the coordinated contraction of heart and smooth muscle [2,8,10,14,15,16], processes that involve dynamic changes in cellular signaling and rearrangement of these subcellular structures. While we still largely lack an understanding of the causality and molecular events connecting these processes to gap junction function, it is increasingly clear that both channel dependent and independent functions drive these processes [17,18,19,20]. Genetic linkage analysis has implicated connexins in at least 28 human diseases—some of which have been recapitulated in mutant connexin mouse models [16,21]. Connexin43 (Cx43), the focus of this study, is by far the most abundantly and widely expressed gap junction protein.

Several reports, both in vivo and in cell culture, indicate that Cx43 has a half-life in the range of 1–3 h [22,23,24,25,26,27,28]—much faster than typical integral membrane proteins (17–100 h) [29,30]. However, the gap junction structure, or gap junction plaque, can exhibit lifespans as long as 20 h (e.g.,[31]). Since Cx43′s normal life cycle includes incorporation into a gap junction followed by degradation ([32] i.e., there does not seem to be a nonjunctional and junctional population of Cx43 with different half lives), gap junctions are in a constant state of flux involving assembly, remodeling, and Cx43 turnover [33,34,35]. Live cell imaging of Cx43-fluorescent protein fusions (Cx43-FP) show that gap junction plaques are highly dynamic exhibiting growth via lateral accretion of connexons into the plaque and fusion of small plaques [36,37,38]. The organization of channels within the plaque can also be quite dynamic but appear to be regulated; there are clear experiments showing organization where the oldest channels can be found in the innermost region of the plaque [36,38] while experiments utilizing fluorescence recovery after photobleaching (FRAP) show that connexon mobility within gap junctions is variable and dependent on the C-terminus [39,40]. Connexon organization within the plaque appears to be regulated through interactions with kinases as treatment with glycyrrhetinic acid altered channel spacing and organization in a manner involving Src kinase [41,42] and PKC [43].

Dynamic interaction with kinases is a key regulator of gap junction biology. We and others have identified >12 phosphorylation sites on Cx43 [24,44,45,46,47,48,49,50,51,52,53,54], and the known Cx43 kinome involves at least PKC, PKA, CK1, ERK1/2, Src, CDC2, Akt, and Tyk2, though some may act indirectly, and multiple additional kinases that phosphorylate Cx43 during key regulatory steps remain to be discovered or more fully validated in vivo. ZO-1 is a well-studied Cx43 interacting partner that has been shown to negatively regulate gap junction size [55], and Akt phosphorylation of Cx43 at S373 (pS373) eliminates ZO-1 interaction and causes a dramatic increase in gap junction size that can be mimicked by the expression of Cx43 with a S373D mutation [56]. Src activity has long been associated with diminished but not complete elimination of gap junctions and inhibition of gap junction intercellular communication. As addressed above, Src can also modulate gap junction organization in a process that involves Src binding [42] and phosphorylation (potentially at Y265) [57], leading to disruption of Cx43-ZO-1 interaction [31,57]. Src has also been shown to interact with ZO-1 and compete for binding to Cx43 [31,58,59].

While maintaining gap junctions in a state of equilibrium is critical to cell and tissue health, stimuli such as growth factors, ischemia, and epidermal wounding lead to rapid clearing of gap junctions from the plasma membrane in a process involving spatiotemporally regulated interaction of connexins with kinases including Akt, MAPK, and PKC [60,61,62]. Gap junction degradation also appears to be a dynamic process involving both the proteasome and lysosome (e.g., [63,64]). Entire gap junction plaques can be internalized by a distinct mechanism where one cell internalizes the entire gap junction resulting in a cytoplasmic double membrane structure termed an annular junction [65,66,67,68,69,70,71,72] or “connexisome” [33]. These structures are clearly visible via electron microscopy, though their abundance varies widely across cell and tissue types, indicating alternate mechanisms exist [60].

Here, we utilize Cx43-FP based model systems in conjunction with lattice light sheet live cell microscopy and airyscan super-resolution microscopy to better understand how gap junction plaques are organized and maintained, how the Cx43 kinome may impact this organization and how different routes of internalization may have consequences on cell behavior. We report that there are phospho-specific subdomains in gap junction plaques (especially phosphorylation at S365 and Y247, a Src site) and saw that formation of connexisomes was associated with activated Src. We observed high levels of MAPK phosphorylation on S279/282 of Cx43 specifically on connexisomes in these cells. We show that Src mediated downregulation of gap junction size and number could be overcome by proteasome inhibition and led to 4.4-fold increase in pY247 levels while pY265, another src site, decreased, arguing that pY247 was specifically preserved within the gap junction. Thus, we propose that there are multiple means of gap junction turnover, and that kinase interactions with Cx43 can drive the method of internalization with phospho-specific subdomains playing a mechanistic role in this process.

## 2. Materials and Methods

### 2.1. Antibodies, cDNA Constructs, and Other Reagents

All general chemicals, unless otherwise noted, were purchased from ThermoFisher Scientific (Waltham, MA, USA). ALLN, MG132, tetradeconoylphorbol acetate (TPA), cycloheximide (CHX), phenylmethylsulfonyl fluoride (PMSF), Brefeldin A (BFA), and a rabbit antibody against Cx43 (C6219) were from MilliporeSigma (Burlington, MA, USA). Mouse anti-Cx43 antibodies, Cx43CT1 and Cx43IF1, were prepared against amino acids 360–382 of Cx43, antibody Cx43NT1 against amino acids 1–20 (Cx43NT1) of Cx43 (described in [49,50,73]), anti-pS368 phosphospecific made to peptide CRPSSRA(pS)SRAS-amide, anti-pS325 made to CQAG(pS)TI(pS)N(pS)HAQP, pY247 phosphospecific made to CKSDP(pY)HATT-amide (see validation of the latter three monoclonal antibodies in Supplementary Figure S1) that we linked via the N-terminal cysteines to maleimide-activated KLH (Pierce Biotechnology, Rockford, IL, USA) according to manufacturer’s instructions and used to generate antibodies at the Fred Hutchinson Cancer Research Center Antibody Technology Facility (Seattle, WA, USA). We made rabbit anti-pY247, pY265, and pS279/282 Cx43 antibodies by custom commercial preparation (ProSci Inc., Poway, CA, USA; 13 week schedule) against synthetic peptides that were phosphorylated at pY247 (CKSDP(pY)HATT-amide), pY265 (Acetyl-KYA(pY)FNGC-amide), and pS279/282 (CAPL(pS)PM(pS)PPGY-amide) linked to KLH as above. Phosphospecific antibody was affinity purified essentially identically to our previously published method [50]. Cx43 fluorescent fusion proteins were generated by cloning Cx43 cDNA into the mammalian expression vectors pENTR4HaloTag (Promega, Madison, WI, USA) and mEmerald-N1 (Addgene, Watertown, MA, USA) via Gibson Assembly cloning. HaloTag ligands AlexaFlour488 and TMR (Promega) were used for imaging.

### 2.2. Cell Line Maintenance and Transfection

Normal rat kidney (NRK) epithelial cells (NRK-E51, American Tissue Culture Collection-ATCC, Manassas, VA, USA), LA-25 cells (NRK cells containing temperature sensitive v-Src [74]) and BWEM cells (a fetal rat myocyte line [75] that expresses Cx43) were cultured in Dulbecco’s Minimal Essential Medium (Mediatech, Pittsburgh, PA, USA) supplemented with 10% fetal calf serum and antibiotics in a humidified 5% CO_2_ environment. Plasmids containing Cx43-FPs were electroporated into cell lines via a Nucleofector apparatus (Amaxa Biosystems, Gaithersburg, MD, USA). Stably transfected clones were isolated with cloning rings in the presence of the selective antibiotic hygromycin (200 µg/mL).

### 2.3. Immunoblotting

For whole cell extracts, cells were lysed in sample buffer containing 50 mM NaF, 500 µM Na_3_VO_4_, 2 mM PMSF, and 1x Complete protease inhibitors (MilliporeSigma) and cellular proteins were separated by SDS-PAGE (10% polyacrylamide). After electrophoresis, protein was transferred to nitrocellulose, the membrane was blocked, and incubated with antibodies to total and appropriate phospho-specific antibody overnight (at 1 µg/mL final concentration) as previously indicated [50]. Blots were incubated with Alexa680-conjugagated anti-mouse and Alexa790-conjugated anti-rabbit secondary antibodies for 1 h and directly quantified using the Li-Cor Biosciences Odyssey IR imaging system and associated software. Normalized densitometry values were calculated as a ratio of phospho-antibody signal over total Cx43 signal.

### 2.4. Immunofluorescence

Cells were washed twice in PBS and fixed in 3.7% formaldehyde for 5 min, permeabilized with 0.5% Triton X-100 and blocked for 30 min in 1% bovine serum albumin in PBS. Cells were incubated with rabbit anti-phosphoCx43 antibody (pY247, pY265) and a total Cx43 anti-mouse antibody (Cx43IF1) or mouse anti-phospho-S279/282 and a total Cx43 rabbit antibody (MilliporeSigma, Burlington, MA, USA) in blocking solution for 1 h. Following several PBS washes, the cultures were incubated with Alexa-488-conjugated donkey anti-mouse antibody and/or Alexa 546-conjugated donkey anti-rabbit antibody for 30–60 min and counterstained with DAPI (Molecular Probes), followed by several washes in PBS and postfixing in 3.7% formaldehyde. The coverslips were mounted onto slides with ProLong Gold Antifade mounting media (ThermoFisher Scientific, Waltham, MA, USA) and viewed with a Nikon Diaphot TE300 fluorescence microscope (Nikon Instruments, Melville, NY, USA) or a Zeiss LSM 780 Confocal microscope (Carl Zeiss Microscopy, White Plains, NY, USA) with Airyscan. Live cell imaging was performed on the lattice light sheet microscope at the Advanced Imaging Core in the Janelia Research Center.

## 3. Results

### 3.1. Live Cell Imaging Shows Multiple Potential Pathways to Gap Junction Turnover

**Figure 1:**
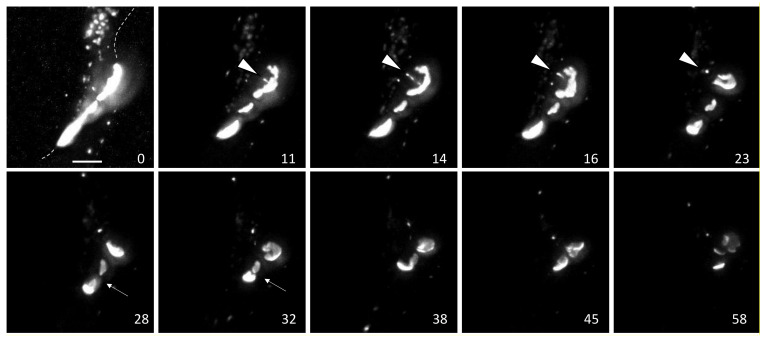
LLSM images from movie of cells expressing Cx43 mEmerald. Time in minutes is indicated in the lower right of each panel and the bar is 5 µm. See movie in Supplementary Materials.

Live cell imaging has provided important insights into gap junction biology while also generating many unanswered questions. In Figure 1, we show data from imaging of Cx43-mEmerald using the lattice light sheet microscope (LLSM) at the Janelia Research Institute, which maximizes spatiotemporal resolution of live cell imaging. Stable cell lines were generated in BWEM cells, a fetal myocyte line [75] that expresses abundant endogenous Cx43 to minimize effects of tagged Cx43 on trafficking and gap junction assembly [37]. While imaging a group of large gap junctions for over an hour, we saw what appeared to be a single, continuous single gap junction that degraded through at least two mechanisms (Figure 1): at minutes 11–23 we observed multiple vesicles leave the center of the plaque in a train-like fashion (arrow in Figure 1) followed by apparent collapse and reorganization of the gap junction plaque (Video S1 available in Supplementary Materials). Over the next 40 min the fluorescence intensity of the plaque diminished greatly, however some areas seemed to maintain a high concentration of connexons (intense white staining similar to starting images) while in other areas fluorescence faded dramatically. These events indicate that specific subdomains in the gap junction can be targeted for an endocytic process while other areas can maintain high concentrations of connexons while other adjacent areas may lose, or at least not replenish, connexons. We also visualized merging of two large plaques over minutes 28–32 followed by shrinking of these structures, but similar to the gap junction above, there was a segregation or perhaps a concentration of an intensely fluorescent subdomain that remained through the course of our timelapse. Note this system has limitations as we cannot distinguish between “new” incoming Cx43 and connexons already residing in the gap junction. For example, the endocytic vesicles may have been a result of segregation of old Cx43 that is targeted for degradation, as has been reported [38]. Nor could we clearly distinguish whether the endocytic vesicles were in fact connexisomes, which would contain connexons from both adjacent cells or whether they contained Cx43 only from their respective parent cells. If they were connexisomes, it is quite intriguing that they exited the plaque as multiple connexisomes; a process that would seem to require a great degree of organization and coordination involving cystoskeletal and associated proteins. We know that phosphorylation regulates interaction of Cx43 with other proteins and can alter gap junction stability, thus kinase interactions at the gap junction could drive formation of specific gap junction subdomains that are targeted for different fates.

### 3.2. Gap Junction Organization and Cx43 Phosphorylation

**Figure 2:**
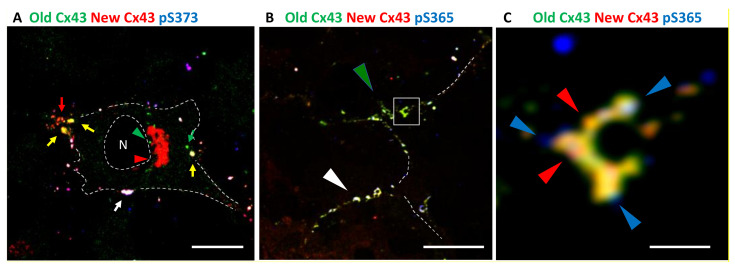
High-resolution Airyscan images of a pulse-chase experiment in NRK cells. Overlays show combined “old” Cx43 as green, “new” Cx43 as red and phosphospecific antibody (pS373 in (**A**) or pS365 in (**B**,**C**)) staining in blue. Thus, white = old+new+ Cx43, pS365 or pY247 Antibody. Yellow = old+new. Purple = new+phosphospecific. C is a higher magnification view of the boxed area in B. Dashed line encircling N indicates the approximate area of the nucleus. Other dashed lines indicate plasma membrane positions. Bar is 10 µm in A and B and 1 µm in C.

To better understand how subdomains could be organized we utilized the HaloTag system to perform pulse chase experiments allowing us to distinguish newly synthesized Cx43 from old and combined this with immunofluorescence using Cx43 phosphospecific antibodies to visualize differential phosphorylation based on the “age” of Cx43 in a gap junction and its organization into gap junction subdomains (see Figure 2). We created stable cell lines expressing Cx43 with a C-terminal HaloTag. We first incubated these cells with the Alexa488 HaloTag ligand that fluoresces green and after an hour washed and incubated with the TMR HaloTag ligand that fluoresces red. Cells were fixed, stained with phosphospecific antibodies (in blue), and imaged using airyscan super-resolution microscopy. Consistent with the reported pathway for Cx43 synthesis and previous results from many groups (e.g., [76,77,78]), we found newly synthesized Cx43 could be seen in the cytoplasm where the staining that appeared could be seen in the cytoplasm where the staining is consistent with its anterograde trafficking through the Golgi Apparatus [79] (Figure 2A, red staining and arrowhead near the nucleus (N)), while most large gap junctions showed a mix of old (green) and new Cx43 (visible as yellow or white, noted by colored arrows). Small, apparently new gap junctions typically lacked green fluorescence and were visible as red or purple, while green only structures could be seen in the cytoplasm representing internalized Cx43 (green arrowheads). Phosphorylation on S373 (pS373) occurred predominantly on gap junctions containing both old and new Cx43, (mostly visible as white, e.g., Figure 2A, white arrow) and, as reported before [56], appeared to occur homogenously on larger gap junctions. In Figure 2B, which includes staining for phosphorylation on S365 (pS365), we see considerable mixing of green and red Cx43 but also do see regions that contain predominantly green/yellow fluorescence (green arrowhead in Figure 2B) while in other areas plaques appear white indicating high levels of pS365 (white arrowhead in Figure 2B). Separate panels for each fluorescent channel are presented in Supplementary Figure S2 along with an overexposed image of Cx43 to help judge cell edges. When we focus in on individual structures (Figure 2C) we begin to see specific subregions that have more intense red fluorescence (red arrowheads) while 365 phosphorylation is quite distinct and concentrated within specific subdomains (blue arrowheads). We clearly observed this phenomenon in complex structures similar to the ones seen in Figure 1 indicating these regions may play a role in dynamic rearrangement and breakdown of gap junctions.

### 3.3. Live Cell Imaging and Mechanisms of Gap Junction Turnover

**Figure 3:**
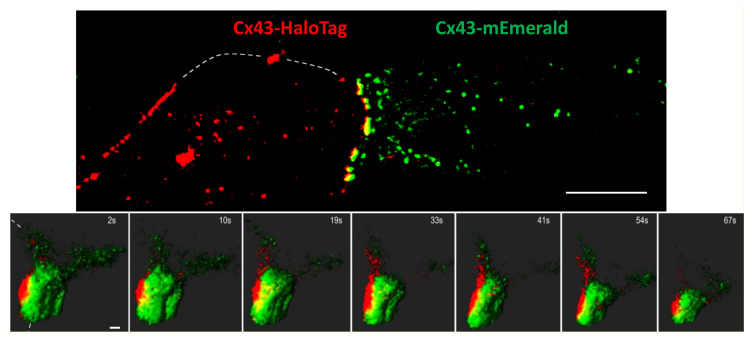
Top panel, LLSM images of BWEM cells expressing Cx43-mEmerald (right cell) mixed with Cx43-HaloTag expressing cells labelled with the red TMR ligand (left cell). Dashed lines indicate approximate plasma membrane positions. Bar is 10 µm. In lower panels, cells were treated with 25 nM TPA to induce disassembly of a large gap junction. Bar is 0.5 µm. See Supplementary Video S2.

To distinguish gap junction degradation via connexisome formation and other means, we mixed cells expressing either Cx43-mEmerald or Cx43-HaloTag labeled with the Janelia Fluor^®^ 647 ligand. As shown in Figure 3, top panel, mixing of these cells results in gap junctions that fluoresce yellow. Our intent was to utilize this system to visualize formation of connexosomes (also known as annular gap junctions) which would be identifiable as yellow cytoplasmic vesicles in our system. Again, we imaged cells on the Lattice Light Sheet microscope which, interestingly, provided enough spatial resolution to visualize the green and red interfaces, in addition to yellow overlap (visible in Figure 3). To our surprise, we saw little to no evidence of connexosome formation after imaging dozens of cells with yellow interfaces for up to 40 min (1 z-stack/2 s) after TPA treatment (Figure 2, see Video S2 in Supplementary Materials). In these BWEM cells, we found that rather than large scale endocytic events driving turnover, most gap junctions would “shrink” (measured after correction for photobleaching) via loss of small, almost exclusively single-color vesicles (0.1–0.5 µm) (Figure 3 and Supplementary Video S2), adding further evidence that multiple routes of disassembly exist. This could have important consequences for cell signaling as internalization of a connexisome would maintain the gap junction structure and its potential role as a scaffold for interacting cytoplasmic proteins, while decoupling it from intercellular communication; on the other hand, the deconstruction of gap junctions and Cx43 internalization we observed in BWEM cells would destroy the scaffold. Regulation of this process could then, both affect and be a reflection of gap junction stability as well as other cell behavior.

### 3.4. Src Regulation of Cx43 Phosphorylation and Gap Junction Turnover

**Figure 4:**
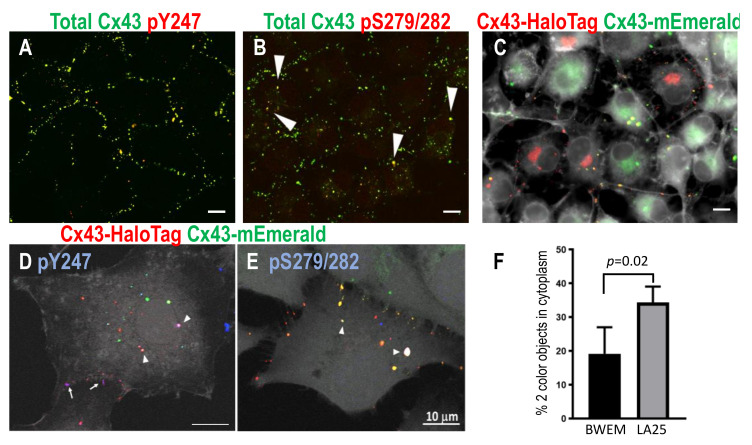
LA25 cells stained for total Cx43 and pY247 (**A**) and pS279/282 (**B**, arrows point to yellow, apparently internalized vesicles or connexosomes). In (**C**–**E**), LA25 cells expressing either Cx43-mEmerald (green) or HaloTag (red) were mixed resulting in gap junctions and connexosomes containing both colors (plasma membrane marker is shown in grayscale). In D, pY247 Ab plus Cx43-HaloTag yields purple indicating phosphorylation of Cx43-HaloTag at gap junctions (arrows) and in connexosomes in cytoplasm (arrowheads) while in E, pS279/282 occurs extensively in connexosomes (arrowheads indicate overlay of green, red, and blue so it appears white). Bars are 10 µm. Quantification, shown in (**F**), shows proportion of Cx43 signal on connexosomes versus gap junctions in BWEM and LA25 cells.

Src is well known to regulate Cx43 and modulate its phosphorylation in a dynamic manner [31,46,80,81]. Src activity leads to a decrease in gap junctions and channel gating and Src directly phosphorylates Cx43 on at least Y247, Y265, and Y313 [80,81,82]. Src activation also increases the activity of multiple other kinases that can and do phosphorylate Cx43 particularly MAPK at residues S279/282 (e.g., [46,83,84]). Immunostaining of LA25 cells (which express temperature-sensitive v-Src and endogenous Cx43) with antibodies to total Cx43 and phosphospecific antibodies to sites phosphorylated by Src (pY247) and MAPK (pS279/282) showed distinct staining where gap junction plaques and large circular cytoplasmic vesicles were positive for pY247 while only the cytoplasmic vesicles were pS279/282 positive (Figure 4A,B, separate panels for each fluorescent channel are presented in Supplementary Figure S3). To determine whether these structures were, indeed, connexisomes, we utilized the Cx43-mEmerald:Cx43-HaloTag approach in LA25 cells to identify bona fide connexisomes. Cells were mixed and allowed to grow for 24–48 h; TMR, a red HaloTag ligand was added, then cells were fixed and stained with AlexaFluor350 phalloidin to mark the plasma membrane (shown in grayscale); yellow objects in the cytoplasm were considered to be connexisomes and were clearly visible in LA25 cells (Figure 4C). Immunostaining with phospho-specific antibodies in this system also showed pY247 (in blue) in both gap junctions and connexisomes while pS279/282 was found at the highest levels in connexisomes (Figure 4D,E, white arrowheads indicate objects showing three color fluorescence as white). Furthermore, quantification of connexisomes in BWEM and LA25 cells showed twice as many in the cells expressing activated Src (Figure 4F). Thus, Src and MAPK may act cooperatively and sequentially to induce connexosome formation. This may not be the only route to connexosome formation, but it does indicate that spatiotemporal interaction of Cx43 with kinases may control the process.

### 3.5. Immunoblot Analysis Indicates Active Src Causes Extensive Changes in Cx43 Phosphorylation

**Figure 5:**
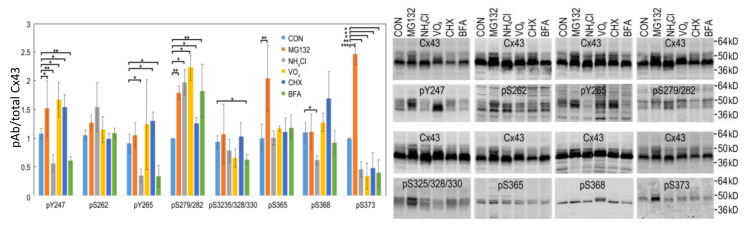
LA25 cells were treated with 10µM MG132, 25 mM NH_4_Cl, 500 µM sodium orthovanadate (Na_3_VO_4_), 25 µg/mL cycloheximide (CHX), or 5 µg/mL Brefeldin A (BFA) for 2 h and immunoblotted for total Cx43 (Cx43) and phosphospecific antibodies for Y247, S262, Y265, S279/282, S325/328/330, S365, S368, and S373. Phospho-antibody signal was normalized to total Cx43 and quantified in graph. Representative immunoblots are shown. *, ** and *** indicate *p* < 0.05, *p* < 0.01 and *p* < 0.001, respectively.

In order to understand the effects of src activation on gap junction turnover, we examined the effects of proteasomal, lysosomal, tyrosine phosphatase, protein synthesis, and trafficking inhibition on LA25 cells. In Figure 5, we show immunoblots of LA25 cells treated with inhibitors for 2 h using phosphospecific antibodies to Cx43 phosphorylated at Y247, S262, Y265, S279/282, S325/328/330, S365, S368, and S373.

Cx43 present in gap junctions has been reported to be turned over by both the proteasome and lysosome [63,85,86,87]. Inhibition of the proteasome for 1–4 h (equivalent to 0.5–2 Cx43 half-lives) causes an increase the amount of Cx43 present in gap junctions with little change in total Cx43 while lysosomal inhibition can cause modest increases in total, mostly nonjunctional Cx43 that runs faster in SDS-PAGE (e.g., [35]), as also shown here in Figure 5 (also shown grouped by inhibitor in Supplementary Figure S5). Proteasomal inhibition had the most dramatic effect on Cx43 phosphorylation leading to a significant increase in several Cx43 phosphorylation events including pY247, pY265, pS279/282, pS365, and pS373. Lysosomal inhibition nearly eliminated phosphorylation by Src on pY247, pY265 while diminishing pS368 and pS373 and increasing pS279/282 levels. Tyrosine phosphatase inhibition (Na_3_VO_4_) increased phosphorylation on pY247 and pS279/282 with no significant change in pY265 while decreasing pS373. Inhibition of protein synthesis had more modest effects in general but increased levels of pY247, pY265, and pS279/282 but decreased pS373. BFA decreased pY247, pY265, pS325/328/330, and pS373 but increased pS279/282. These data exemplify the complex spatiotemporal regulation of Cx43 and gap junction stability as essentially no sites responded to these inhibitors in concert; even pY247 and pY265, both Src sites, showed differential responses to MG132 and CHX. Interestingly, MAPK phosphorylation on S279/282 was increased under all conditions indicating that interaction with MAPK may be quite promiscuous occurring at multiple subcellular locations while phosphorylation on S373 by Akt was diminished in all cases except proteasome inhibition, consistent with Akt mediated phosphorylation of Cx43 occurring only in the gap junction itself (also shown in Figure 2A and [56]).

**Figure 6:**
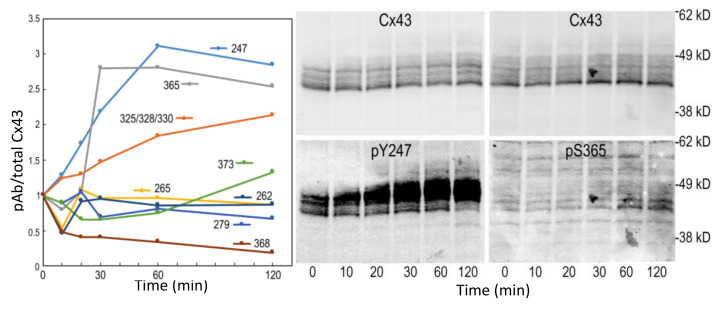
Time course of the change in Cx43 phosphorylation changes in response to MG132 treatment. The blots for the sites exhibiting the largest change are shown (pY247 and pS365).

Since proteasomal inhibition had the largest effect, we performed a series of MG132 treatments to determine how Cx43 phosphorylation changed over 2 h (Figure 6). Indeed, we saw the biggest changes in phosphorylation occurred by 60 min of treatment, with the most dramatic changes in phosphorylation on pY247 and pS365.

### 3.6. Proteasomal Inhibition Blocks the Effects of Src Activation on Gap Junction Size

**Figure 7:**
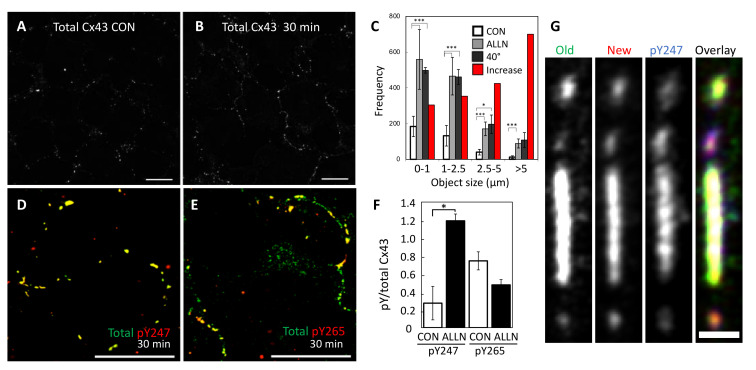
Immunofluorescent staining of LA25 cells for total Cx43 (Cx43IF1) in the absence (**A**) or presence of a proteasomal inhibitor (**B**) and the resulting quantification of junction size (**C**) comparing control to the proteasomal inhibitor ALLN (red bars indicate the percent increase elicited by proteasome inhibition) and cells incubated at 40 °C (src inactive). In (**D**,**E**), cells were stained for total Cx43 (green, Cx43IF1) and pY247 (D, red) or pY265 (E, red), resulting overlay is yellow, and the ratios are indicated in (**F**). G shows a higher magnification view of a large gap junction showing old, new, and pY247 subdomains. A, B, D, F Mag bars are 20 µm, G is 1 µm. * and *** indicate *p* < 0.05 and *p* < 0.001, respectively.

To determine whether the dramatic changes in the phosphoprofile of Cx43 translated to changes in Cx43 localization, we examined Cx43 immunofluorescence in LA25 cells treated with MG132 and found a dramatic increase in both gap junction number and gap junction size (Figure 7A–C). This shift occurred rapidly and was clearly visible with 30 min of proteasome inhibition (Figure 7A,B). Interestingly, this new distribution of Cx43 into apparent gap junctions almost exactly mimicked the number and size of gap junction in cells where Src was not active (Figure 7C, 40 °C causes inactivation of src in these cells). This suggests that an, as yet unknown, proteasome sensitive factor may actually play a major role in Src mediated downregulation of gap junctions. Similar to what we saw by immunoblot, we found that pY247 was dramatically increased while pY265 actually showed lower levels that were not significantly different than control (Figure 7D–F, Supplementary Figure S4 shows individual channels at lower magnification for better context). Indeed, when we focused in on individual gap junctions using the pulse-chase approach in LA25 cells expressing Cx43-HaloTag, we saw that the oldest Cx43 was more homogenously distributed through a large gap junction and also visible in small vesicles that appeared to be exiting the large plaque (Figure 7G). We also saw segregation of Cx43 into smaller gap junctions that fluoresced strongly in the green channel (uppermost plaque) while others fluoresced strongly in the red channel (bottom plaque). Similar to what we observed for pS365, pY247 was concentrated into distinct subdomains of the large gap junction and quite low in the topmost and bottom gap junctions. It is worth noting that in addition to showing a strong subdomain localization, both pS365 and pY247 were strongly preserved by proteasomal inhibition; consistent with these events playing a mechanistic role in gap junction turnover.

## 4. Discussion

Gap junction stability is a highly regulated process that involves interaction of a variety of proteins, cytoskeletal elements, and signaling pathways many of which have been well described previously (e.g., [20,88]). These interactions combined with evidence for channel independent functions for Cx43 are consistent with gap junctions providing a scaffold function in cells. The short half-life of Cx43 and dynamic nature of these structures provide a means for cells to rapidly communicate and respond to changes through both extracellular or intracellular signaling via responses to stimuli including oncogenes, growth factors, or ischemia and also through intercellular means via gap junction channel function. Organization of gap junctions into subdomains could create signaling nodes for cytoplasmic interacting proteins, contribute to segregation of old and new channels and flux in stable gap junctions, and provide points of interaction for the cytoskeleton and endocytic proteins. Segregation of old and new gap junction channels has also been shown previously using the FLAsH/ReAsH system [38] and photoconvertible proteins [89]. Here, we show an endocytic process where multiple vesicles leave a gap junction (Figure 1, Video S1). The apparent collapse and rearrangement of the plaque immediately following this event leads us to hypothesize that a change in either the arrangement of connexons to a more fluid organization or an interaction with the cytoskeleton may have occurred. Other studies utilizing FRAP [39,40] have shown that connexon mobility is regulated in a manner dependent on the C-terminus of Cx43 (perhaps due to phosphorylation) and gap junction stability is known to involve interactions with microtubules, the clathrin cytoskeleton and the actin cytoskeleton. Thus, endocytosis may not regulate only degradation but also gap junction organization and fluidity.

In the second video (Video S2), we show a large gap junction that reduced in size mainly through loss of many single color vesicles to the cell from which it originated. In Figure 4 we show that src activity can enhance connexisome formation; as a whole these data argue that at least two pathways to gap size reduction exist and that they can exist in a single cell. Using super-resolution microscopy, we show that Cx43 phosphorylated on S365 and Y247 is able to cluster into subdomains, while pS373 appears to occur throughout the plaque, indicating regulation of phospho-specific subdomains. This finding that specific phosphorylation sites form subdomains could provide a mechanistic point of interaction with the cytoskeleton or provide tethers for kinase interaction and organization. This organization coupled with the upregulation of pY247 and pS365 with proteasome inhibition supports the idea that these sites are involved in internalization.

The phospho-profile of gap junctions compared to connexisomes, as shown in Figure 4, indicate kinases play a role in directing disassembly pathways. In this system, we see that connexisomes have high levels of MAPK phosphorylation combined with Src phosphorylation on Y247. As reported by several other groups the MAPK sites overlap a PY motif (S282-Y286) that mediates interaction with NEDD4, a ubiquitin ligase. Downregulation of NEDD4 leads to increased gap junction stability [90,91,92,93] and phosphorylation on S279/282 has been shown to stabilize Cx43-NEDD4 interaction [93,94]. In addition, there are two tyrosine sorting-motifs at residues Y286-V289 and Y265-F268 [95,96]; these motifs promote sorting of membrane proteins to endosomes and lysosomes via interaction with adaptor protein complexes [97]. Several groups have studied how these domains control interaction with clathrin [98,99,100]. In one system utilizing cells that express Cx43 but do not make gap junctions, connexons were endocytosed from the plasma membrane prematurely via clathrin mediated endocytosis. However, this could be overcome by mutation of the tyrosine sorting signal or the MAPK sites S279/282 [67]. Interestingly, the sorting motif mutation led to connexisome formation in a clathrin-independent manner. Our data showing pS279/282 on connexisomes is consistent with the idea that this phosphorylation event obscures the sorting motif and plays a role in driving connexisome formation. In addition, phosphorylation on Y265 would likely obscure the sorting motif at Y265-F268 enhancing this effect. Thus, in agreement with several reports [31,81,101,102] src activation promotes connexosome formation.

Our finding that treatment with a few of the inhibitors in our panel could dramatically and specifically alter the phospho-profile of Cx43 illustrates the complex interactions involved in gap junction regulation. While caution must be exercised when attempting to interpret the results from any inhibitor study, what was clear was that each inhibitor led to a unique phosphoprofile (Figure 5 and Supplementary Figure S5), consistent with a high level of spatiotemporal regulation of Cx43. For example, lysosome/autophagosome inhibition via NH_4_Cl increases ERK site phosphorylation while inhibiting src phosphorylation. On the other hand, inhibition of tyrosine phosphatases with vanadate increased the phosphorylation levels of Y247 and S279/282 in tandem. Whether this is due to direct effects on Cx43 or changes in src signaling is not clear at this time. Particular caution is necessary for interpreting protein degradation inhibitor data; proteasome inhibition has effects on many short-lived proteins that may be playing a role in regulating gap junction stability. Remarkably, we saw that proteasome inhibition could effectively counteract the effects of src activation on gap junction size and do so quite rapidly (Figure 7), arguing that src activity could destabilize a proteasome sensitive factor involved in gap junction stability. This linkage between proteasome activity and gap junction stability appears to involve a specific role for S373 phosphorylation as indicated by the fact this site was downregulated in all conditions except proteasome inhibition. One interpretation of increases in specific Cx43 phosphorylation sites caused by proteasomal inhibition is that these sites are involved in the mechanism of degradation and thus, specifically preserved when gap junctions grow. Phosphorylation on both S365 and S373 were uniquely increased in response to proteasome inhibition (Figure 5) arguing these sites may link proteasome activity to gap junction stability. However, it is more complicated as we also observed increased pY247, pY265, and pS279/282 sites typically associated with downregulation of gap junctions (Figure 5). Proteasome inhibition has been previously shown to stabilize gap junction formation in other cell types as well (e.g., [103,104]). Typically, these cells show a lack of phosphorylation on S365 and CK1 sites which are associated with gap junction stability [32,47,50]. We have previously shown, in unstimulated cells, that proteasome inhibition leads to increased interaction with and stabilization of Akt, phosphorylation on S373, inhibition of ZO-1 interaction, and plaque growth [56]. The increase in pS373 shown in Figure 5 is consistent with this effect but given that ZO-1 is likely already displaced in this system and the potential widespread effect of proteasomal inhibition, other factors or constraints likely play a role in limiting plaque size.

**Figure 8:**
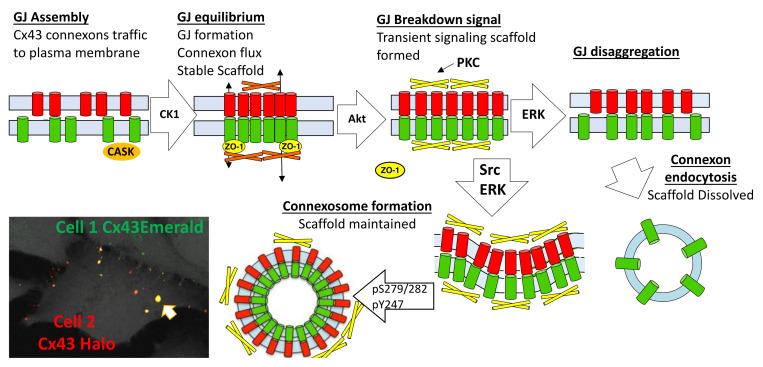
Model incorporating some known effectors for Cx43 life cycle and gap junction assembly and turnover. Arrow in left bottom indicates a yellow internalized connexosome.

In Figure 8, we present a model for the gap junction lifecycle. Cx43 traffics to the plasma membrane as a connexon or hemichannel. En route to the plasma membrane Cx43 interacts with CASK [52], a scaffold protein, then interacts with ZO-1 in the plasma membrane and in the gap junction plaque [55,105,106]. Phosphorylation by CK1 appears to stabilize Cx43 in gap junctions and a homeostatic scaffold is formed. Gap junction and scaffold equilibrium is maintained through flux of Cx43 in and out of the gap junction. Various stimuli induce acute remodeling of gap junctions that include phosphorylation by Akt and consequent growth of the gap junction. Due to a change in interacting proteins, this scaffold will have different signaling properties than the homeostatic scaffold and, thus, will contribute to how cells respond to stimuli. Subsequent phosphorylation events involving MAPK, PKC, and Src lead to disassembly of the gap junction. Differential phosphorylation of Cx43 can lead to different pathways of disassembly. A model where the gap junction falls apart, through disaggregation and internalization would eliminate both channel and scaffold function from cells. Formation of a connexosome, on the other hand, could maintain the scaffold formed in response to stimuli thereby enhancing and prolonging this signaling. Acute disassembly is often also accompanied by inhibition of anterograde Cx43 trafficking [24] leading to a temporal lag in resumption of gap junction signaling through the homeostatic scaffold. Thus, different pathways of disassembly could dramatically alter Cx43 mediated effects on cell behavior.

Chronic inhibition of gap junctions through post-translational means is a common feature of tumor cells [107] and is seen in a variety of cardiac and other pathologies [108,109]. Src activation in LA25 cells, shown here, is particularly interesting in that it has an intermediate phenotype, where cells can make gap junctions but they are smaller and less abundant than when Src is not active (Figure 7C) and channels are gated [46,82,83,110]. Src is known to competitively bind to and remove ZO-1 from the scaffold priming cells for PKC phosphorylation [59,111]. The phospho-profile of Cx43 in Src activated cells is similar to that seen during acute disassembly [46,60,112]; thus, Src interaction with Cx43 may stabilize a signaling scaffold that is normally only transiently active. Furthermore, these cells are very efficient at making connexisomes (Figure 4F) further amplifying this signaling and uncoupling it from intercellular communication. We propose this represents a mechanism by which oncogenes could hijack gap junction signaling.

## 5. Conclusions

Using state-of-the-art live and super resolution imaging methods, specific inhibitors and probably the largest collection of phosphorylation-status specific antibodies assembled, we found phospho-specific domains in gap junction plaques and connexosomes and show evidence that multiple pathways of disassembly exist that can be regulated at the cellular and subcellular level and act within what appears to be a single gap junction. We also report the formation of specific phospho-specific subdomains in a plaque that could mediate interactions with the cytoskeleton and affect gap junction structure. The stability of gap junctions affects signaling through both channel and scaffold functions but how these are integrated is not well understood. Here we suggest a model where different routes of gap junction disassembly actually regulate the integration of these functions through different, but not mutually exclusive, scenarios including: (1) homeostatic cells maintain stable gap junctions that engage in intercellular communication and serve as a scaffold for homeostatic signaling that can be rearranged through interaction with kinases in response to specific cues; (2) disaggregation and internalization of connexons into their parent cells downregulates both gap junction channel and scaffold function; (3) formation of connexisomes maintains scaffold function while separating it from channel function. This concept of differential scaffold functions and altered spatiotemporal regulation of these scaffolds by kinases adds to our understanding of how Cx43 can regulate cell behavior. Our finding that Src can actually hijack aspects of these processes offers insight into how post-translational modification of Cx43 may be targeted in a variety of pathologies to alter cellular signaling.

## Supplementary Materials

The following are available online at https://www.mdpi.com/2218-273X/10/12/1596/s1, Figure S1: Validation of specific monoclonal antibodies, Figure S2: Individual fluorescence channel signals for Figure 2A–C, Figure S3: Individual fluorescence channel signals for Figure 4A,B, Figure S4: Individual fluorescence channel signals for Figure 7D,E, Video S1: Unique pathways for gap junction disassembly, Video S2: Reduction in gap junction size through loss of vesicles to their cell of origin.
