# Supplementary material for: Src Regulation of Cx43 Phosphorylation and Gap Junction Turnover
Source: Biomolecules. 2020 Nov 24;10(12):1596. doi: 10.3390/biom10121596 (PMC7759836; doi:10.3390/biom10121596)

Supplemental Figure S1 – monoclonal antibody validation

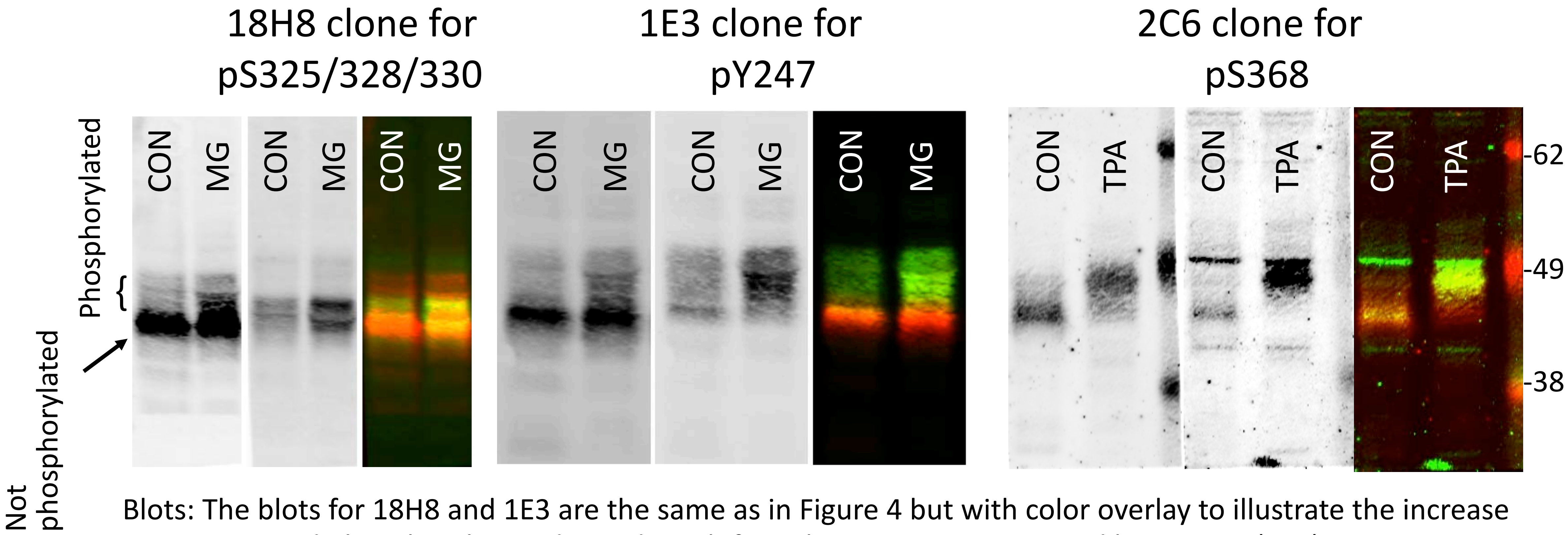

Blots: The blots for 18H8 and 1E3 are the same as in Figure 4 but with color overlay to illustrate the increase in intensity and phosphorylation dependent shift to slower migration caused by MG132 (MG) treatment. For pS368 we did not see much of a shift or increase by MG123 treatment, so we utilized CHO cells untreated or treated with TPA to increase phosphorylation on Cx43 at serine 368 and decrease migration. In the right panel the phosphorylation level is shown to be increased.

Immunofluorescence: Below the antibodies are used in immunofluorescence (green in Cx43, phosphospecific antibodies in red). The lower 4 panels are MDCK cells expressing wild type or mutant Cx43.

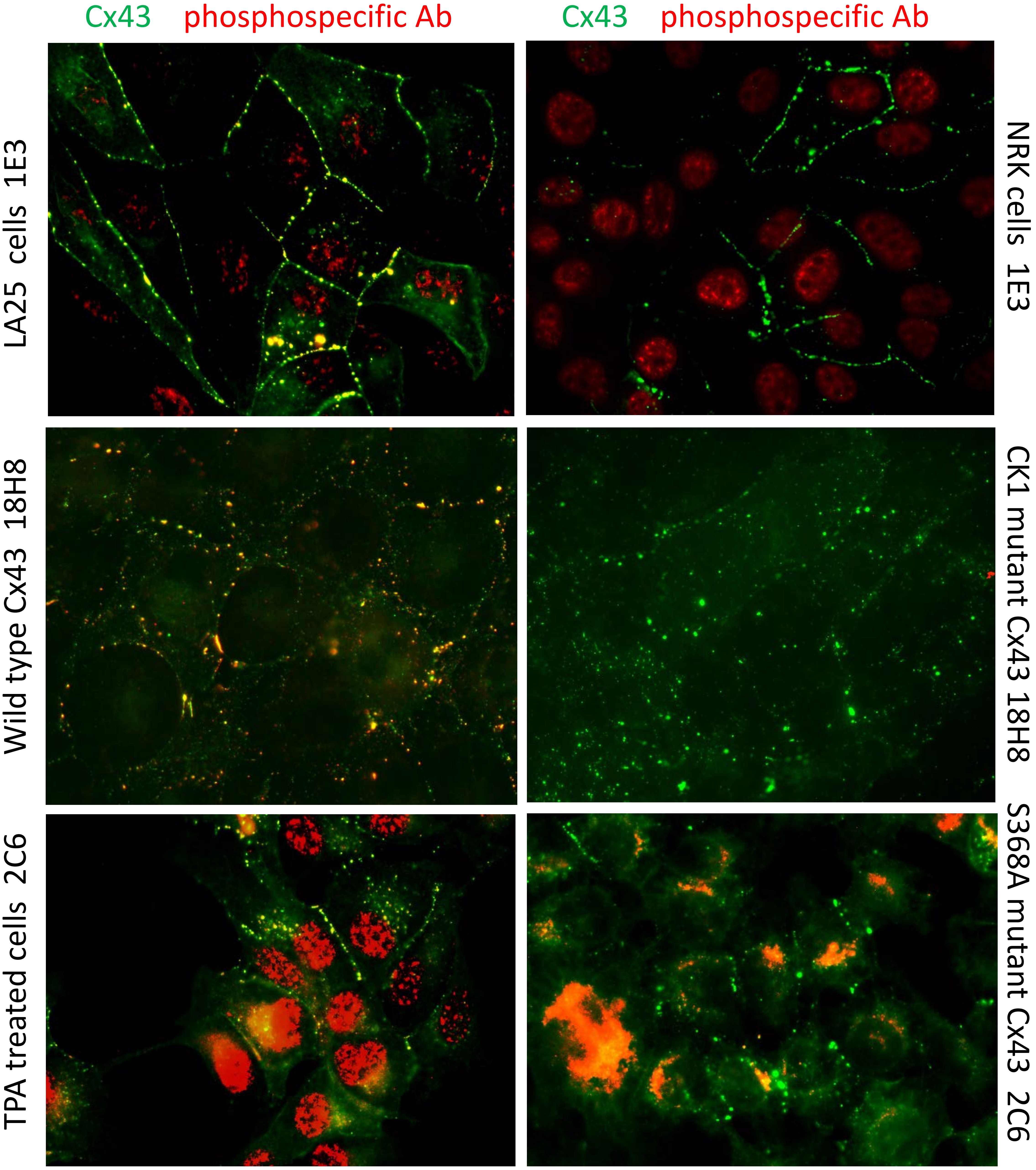

Supplemental Figure S2, Supports Fig. 2A-C

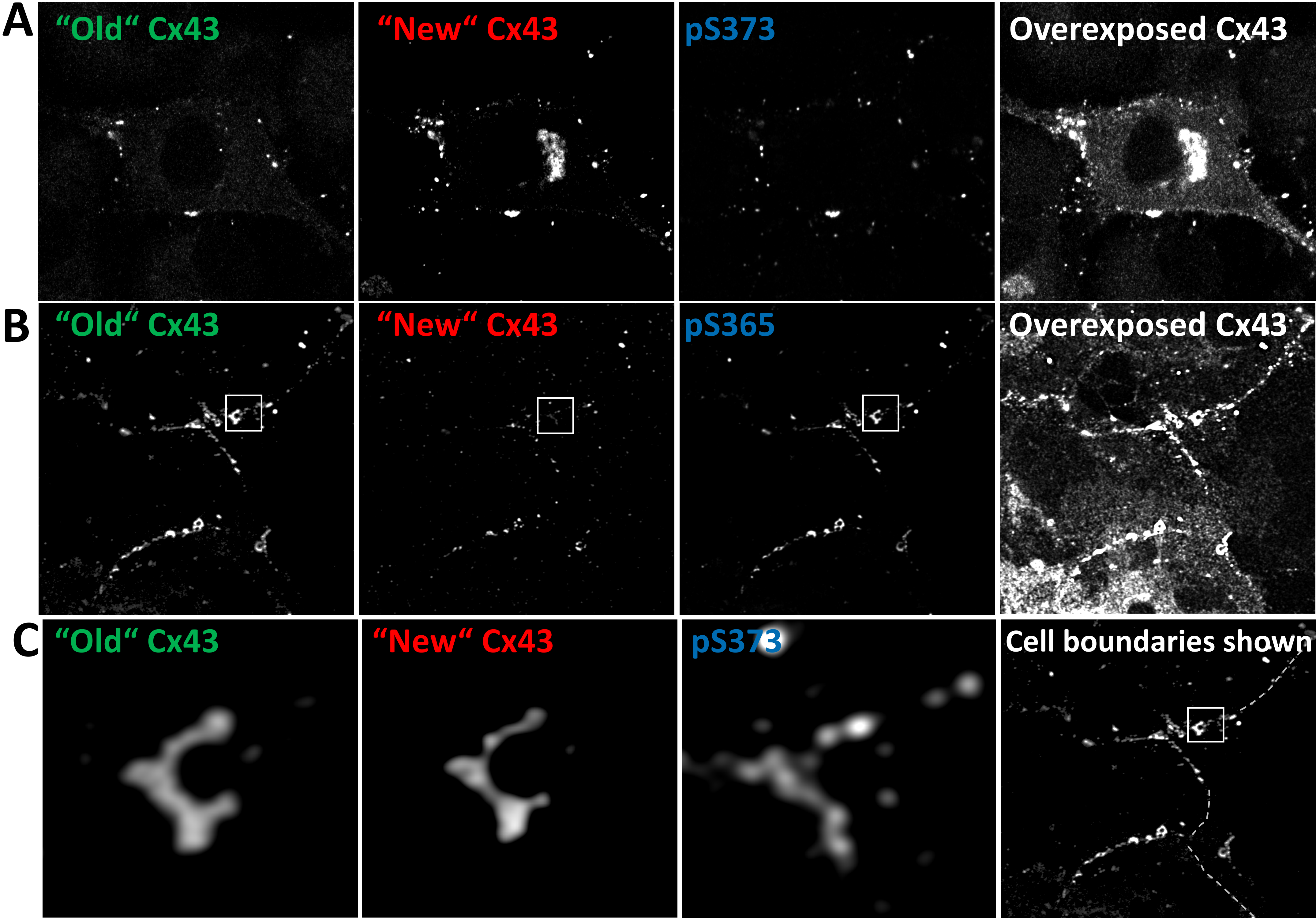

Individual channel signals for Alexa488 HaloTag ligand (green, “old”), TMR HaloTag ligand (red, “new”) and phosphospecific antibodies (blue) for Figures 2 A-C. Overexposed image included to show cell boundaries. Dotted line in last panel of C. also shows cell boundaries denoted by dashed line.

Supplemental Figure S3, supports Figure 4A and 4B

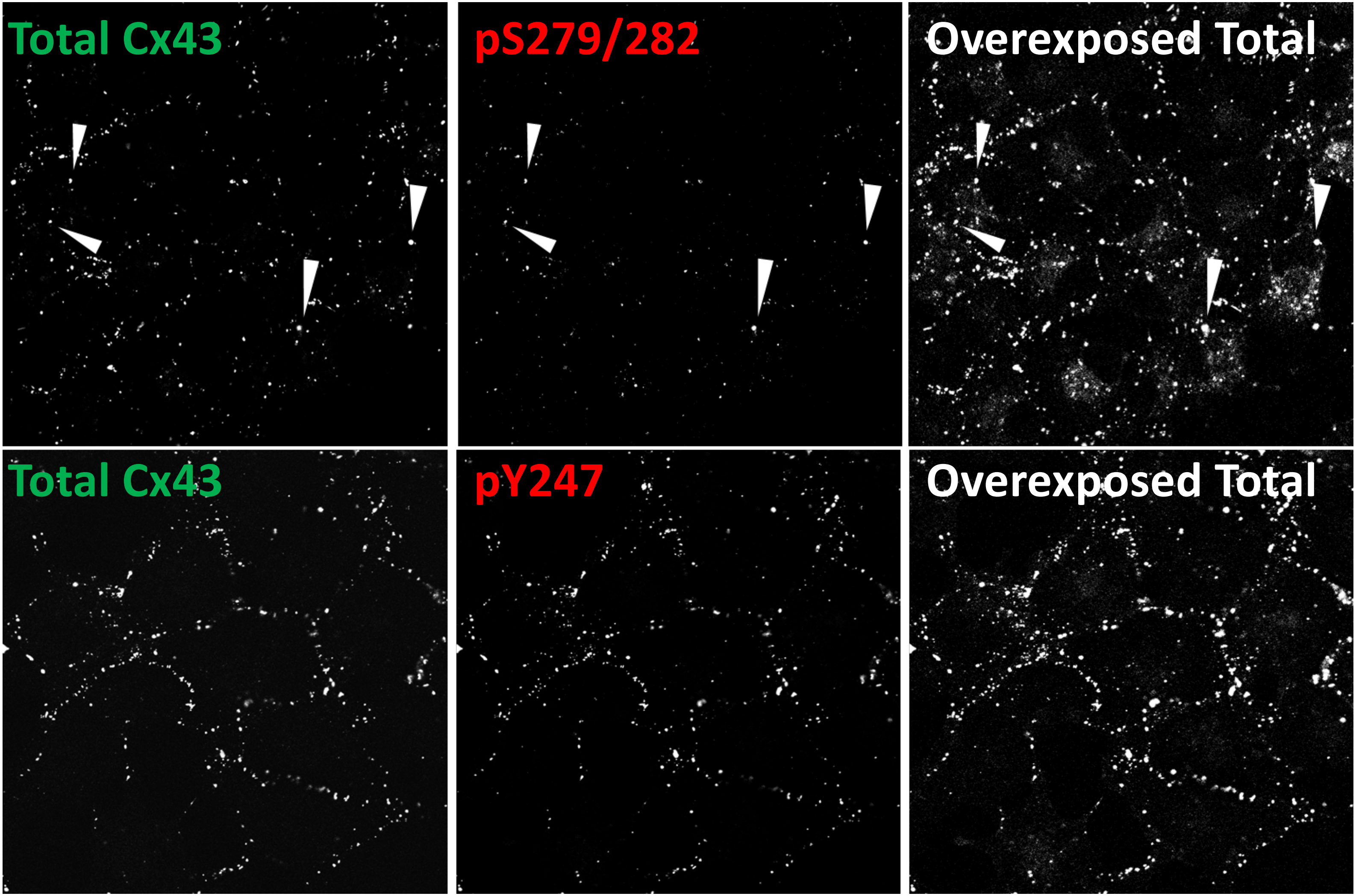

Individual channel signals for Total Cx43 (green) and phosphospecific antibodies (red) for Figures 4A and 4B. Overexposed image included to show cell boundaries.

Supplemental Figure 4, supports Figure 7D and E

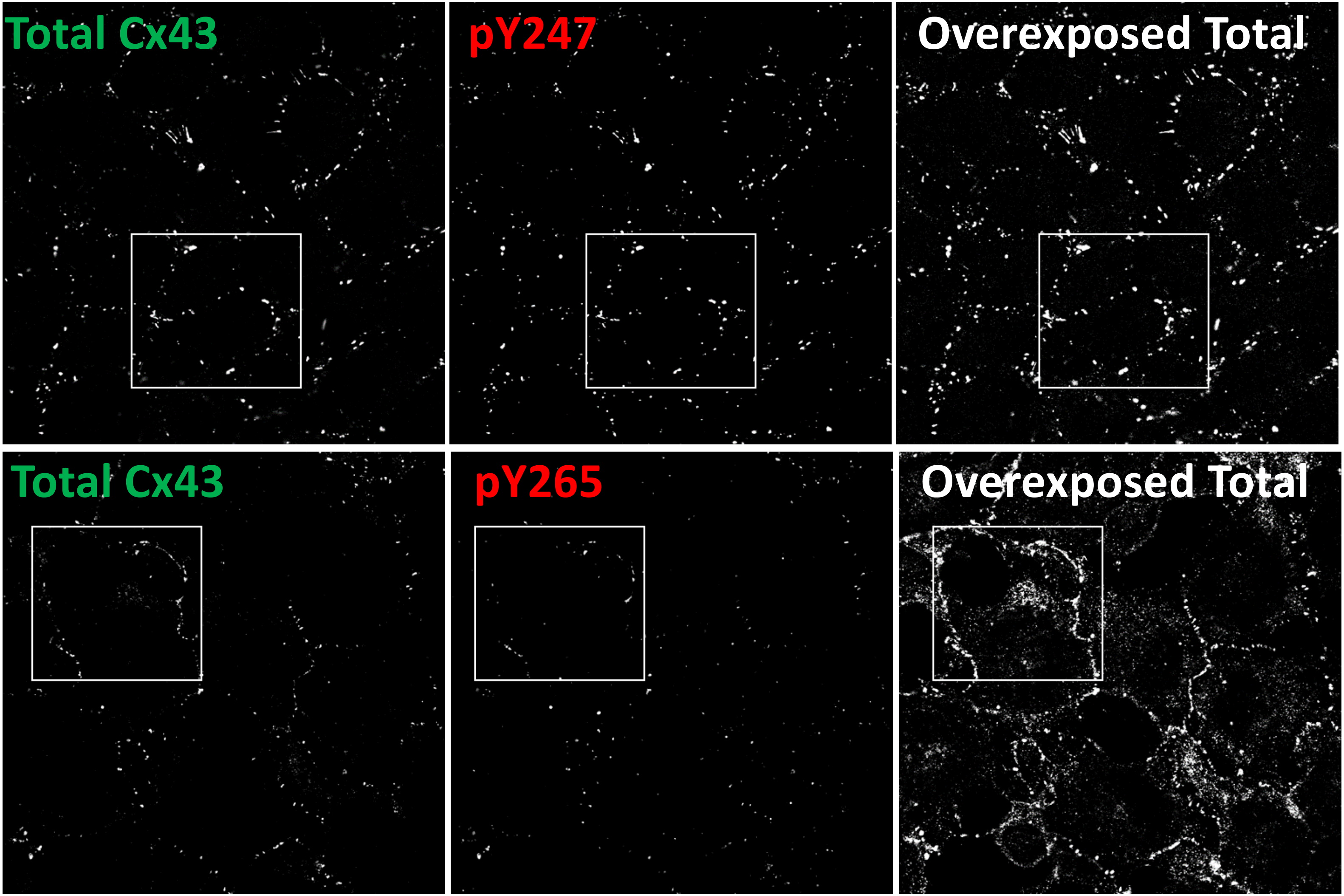

Individual channel signals for Total Cx43 (green) and phosphospecific antibodies (red) for Figures 7D and 7E. Overexposed image included to show cell boundaries.

Supplemental Figure S5, supports Figure 5

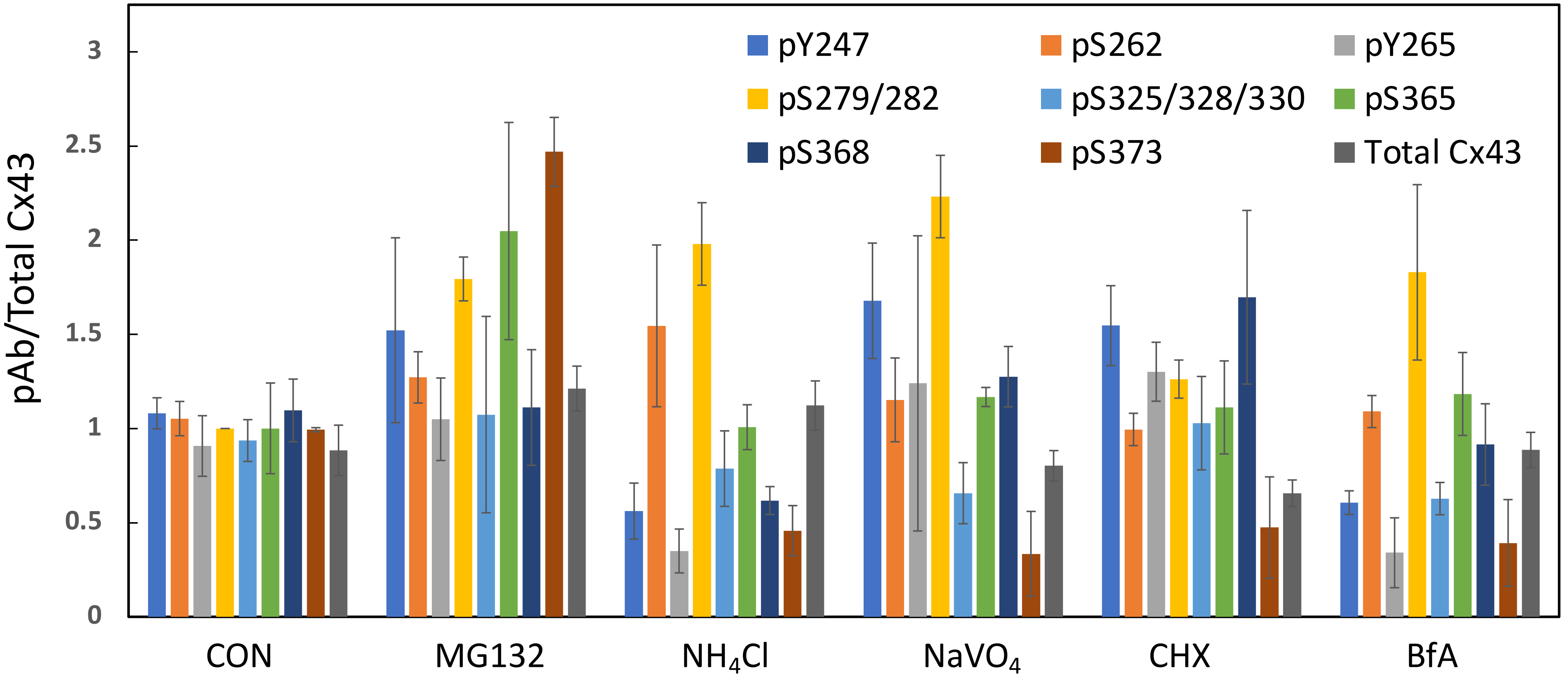

Supplement: Supplementary file 1 [file biomolecules-10-01596-s001.zip › biomolecules-996614 supplementary revised.pdf]
